# Ruxolitinib reverses systemic vasculitis driven by JAK2 V617F–mutated essential thrombocythemia: a case report

**DOI:** 10.3389/fimmu.2026.1781998

**Published:** 2026-03-13

**Authors:** Yutaro Ashida, Shinji Ota, Nao Ishidoya, Yasuhisa Murai, Kyoko Amenomori, Keisuke Hasui, Shogo Kawaguchi, Satoko Yamaguchi, Hiroto Hiraga, Kosuke Kamata, Hiroaki Ichikawa, Hirofumi Tomita, Zaiqiang Yu, Masahito Minakawa, Hirotake Sakuraba

**Affiliations:** 1 Department of Gastroenterology, Hematology, and Clinical Immunology, Hirosaki University Graduate School of Medicine, Hirosaki, Japan; 2 Department of Vascular and Inflammatory Medicine, Hirosaki University Graduate School of Medicine, Hirosaki, Japan; 3 Department of Bioscience and Laboratory Medicine, Hirosaki University Graduate School of Health Sciences, Hirosaki, Japan; 4 Department of Cardiology and Nephrology, Hirosaki University Graduate School of Medicine, Hirosaki, Japan; 5 Department of Thoracic and Cardiovascular Surgery, Hirosaki University Graduate School of Medicine, Hirosaki, Japan

**Keywords:** JAK2 V617F mutation, myeloproliferative neoplasms, ruxolitinib, thrombocythemia, vasculitis

## Abstract

**Background:**

Vasculitis associated with myeloproliferative neoplasms is rare, and reports on its treatment are limited. Herein, we report a case of *Janus Kinase2 (JAK2)* V617F mutation–positive essential thrombocythemia (ET) associated with vasculitis.

**Case presentation:**

A 60-year-old man with *JAK2* V617F mutation–positive ET underwent coronary artery bypass grafting after recurrent myocardial infarctions and coronary stent occlusions. Intraoperative and pathological findings led to a diagnosis of vasculitis. Treatment with hydroxycarbamide and ruxolitinib resulted in decreased platelet counts and improved vasculitis, with no subsequent recurrence of cardiovascular events.

**Conclusion:**

This rare case shows that ruxolitinib can be effective in treating vasculitis complications in patients with *JAK2* mutation–positive ET.

## 1 Introduction

Myeloproliferative neoplasms (MPNs) are clonal disorders of hematopoietic stem cells that are characterized by excessive myeloid proliferation. *Janus Kinase2 (JAK2)* V617F mutation has been detected in approximately 50% of essential thrombocythemia (ET) cases (1). In addition to hematological abnormalities, the *JAK2* V617F mutation is associated with an increased risk of thrombosis and systemic inflammation via abnormal activation of neutrophils and macrophages, manifesting as thrombosis and increased inflammatory cytokine production (1–3). The combination of MPNs and vasculitis is rare, and standard treatment guidelines have not yet been established. A French multicenter retrospective case–control study reported that giant cell arteritis (GCA) complicated by *JAK2* V617F mutation–positive MPN responds to glucocorticoids but has a poorer prognosis than isolated GCA, with cardiovascular events and infections being common causes of death (4).

JAK inhibitor therapy is expected to be clinically beneficial for JAK2 V617F mutation-positive MPN with vasculitis, however, reports on this are limited. Here, we report a case in which ruxolitinib effectively improved systemic vasculitis complicating *JAK2* V617F mutation–positive ET.

## 2 Case description

A 60-year-old man with a medical history of left cortical cerebral infarction and percutaneous coronary intervention for unstable angina was receiving treatment for hypertension, dyslipidemia, and diabetes at another hospital. He was taking oral antithrombotic medications: aspirin 100 mg/day, prasugrel 3.75 mg/day, and warfarin 3 mg/day. He had been smoking 60 cigarettes per day for the past 27 years. Because the patient experienced recurrent exertional chest pain since August 2024, he visited the cardiology department of our hospital in November 2024.

In December 2024, the patient underwent percutaneous coronary intervention for unstable angina (left anterior descending artery). The following day, stent thrombosis was detected and thrombus aspiration was performed. Despite concomitant antiplatelet therapy, stent restenosis was observed 2 weeks later. Catheter-based intervention was deemed difficult, and coronary artery bypass grafting was performed. Intraoperatively, macroscopic findings revealed full-thickness of wall with severe inflammation and intimal thickening in the coronary artery. Furthermore, the right internal thoracic artery exhibited easy dissection and demonstrated fragility. Additionally, the patient presented with elevated white blood cell and platelet counts at admission, which led to a referral to our department.

Physical examination revealed no fever, jaw claudication, or temporal artery tenderness. Soft white spots were observed in both the ocular funduses. Laboratory tests showed the following results: white blood cell count, 14,340/μL (Neutrophils 80%, Lymphocytes 8%, Monocytes 6%, Eosinophils 2%, Basophils 3%); platelet count, 65.1×10^4^/μL (giant platelets, varying in size); lactate dehydrogenase, 1,002 U/L; C-reactive protein, 88.6 mg/L; ferritin, 759 ng/mL; erythrocyte sedimentation rate, 40 mm/h; antinuclear antibody, negative; proteinase 3-anti-neutrophil cytoplasmic antibody, negative; anti-glomerular basement membrane body, negative; myeloperoxidase-anti-neutrophil cytoplasmic antibody, negative; blood glucose, 239 mg/dL; glycated hemoglobin, 7.1%; *JAK2* V617F variant allele frequency(VAF) in peripheral blood was 60.7%; *MPL* mutations, negative; *CALR* mutations, negative; *JAK2* exon 12 mutations, negative; *BCR: ABL1*, negative.

**Figure 1:**
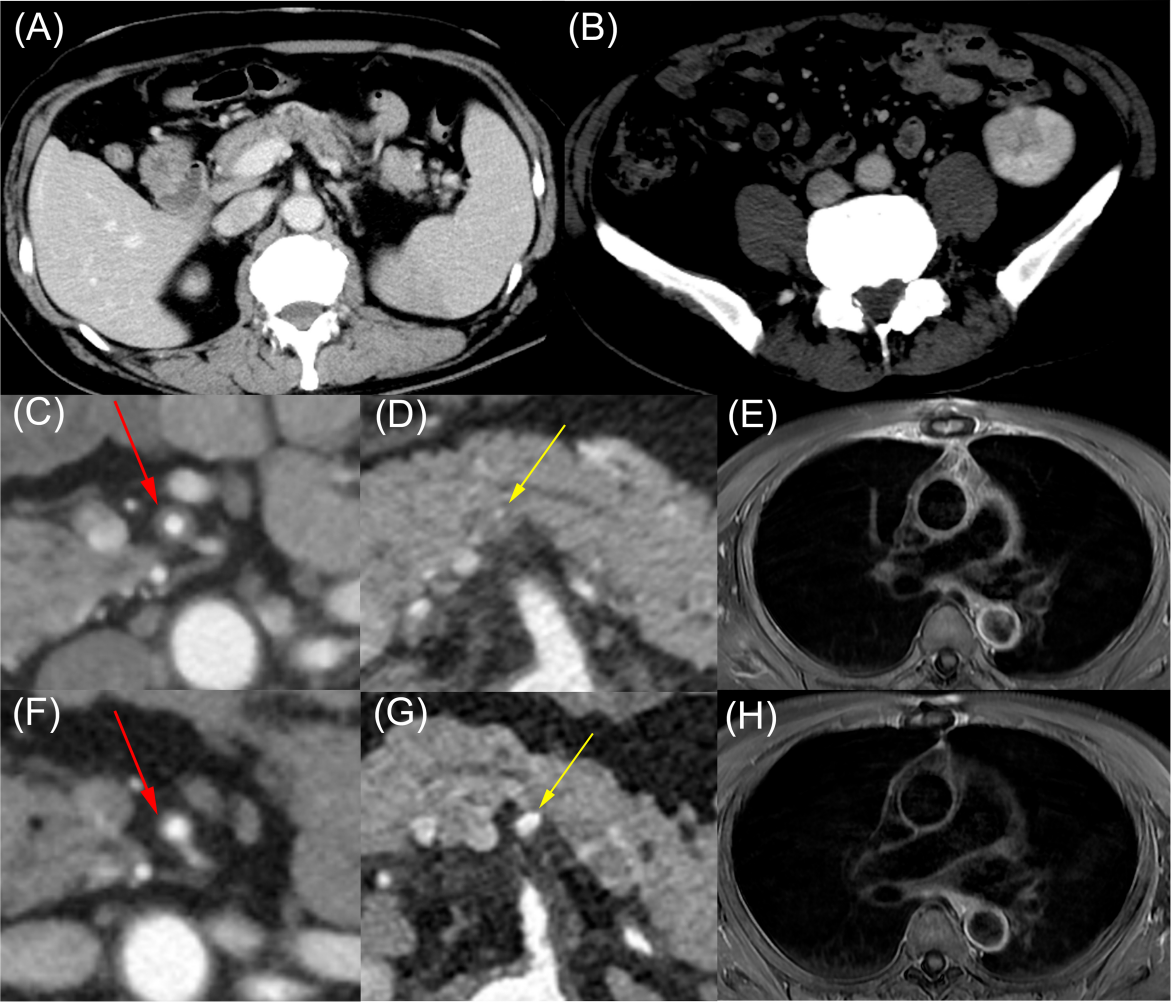
Imaging findings of *JAK2* V617F-positive essential thrombocythemia with multivessel vasculitis before and after ruxolitinib therapy. Contrast-enhanced computed tomography (CT) and magnetic resonance imaging (MRI) revealed visceral infarctions and systemic vasculitis before treatment, with marked improvement after ruxolitinib therapy. **(A, B)** Contrast-enhanced CT showing splenic infarction **(A)** and partial left renal infarction **(B)**. **(C–E)** Pretreatment contrast-enhanced CT and MRI demonstrating vasculitic changes in medium- and large-sized vessels, including wall thickening with stenosis of the superior mesenteric artery [**(C)**, red arrows], wall thickening with stenosis at the origin of the splenic artery [**(D)**, yellow arrows], and vascular wall thickening with contrast enhancement of the ascending and descending aorta **(E)**. **(F–H)** Follow-up contrast-enhanced CT and MRI obtained 6 months after ruxolitinib therapy demonstrated marked improvement in vasculitic changes in the superior mesenteric artery [**(F)**, red arrows], the origin of the splenic artery [**(G)**, yellow arrows], and the aorta **(H)**.

Computed tomography demonstrated infarctions in the spleen and left kidney (**Figures 1A, B**), along with wall thickening and stenosis involving the superior mesenteric artery and the origin of the splenic artery (**Figures 1C, D**). Magnetic resonance imaging revealed vascular wall thickening and enhanced contrast in the ascending aorta, aortic arch, descending aorta, left common carotid artery, and left subclavian artery (**Figure 1E**). Positron emission tomography–computed tomography showed no significant uptake.

**Figure 2:**
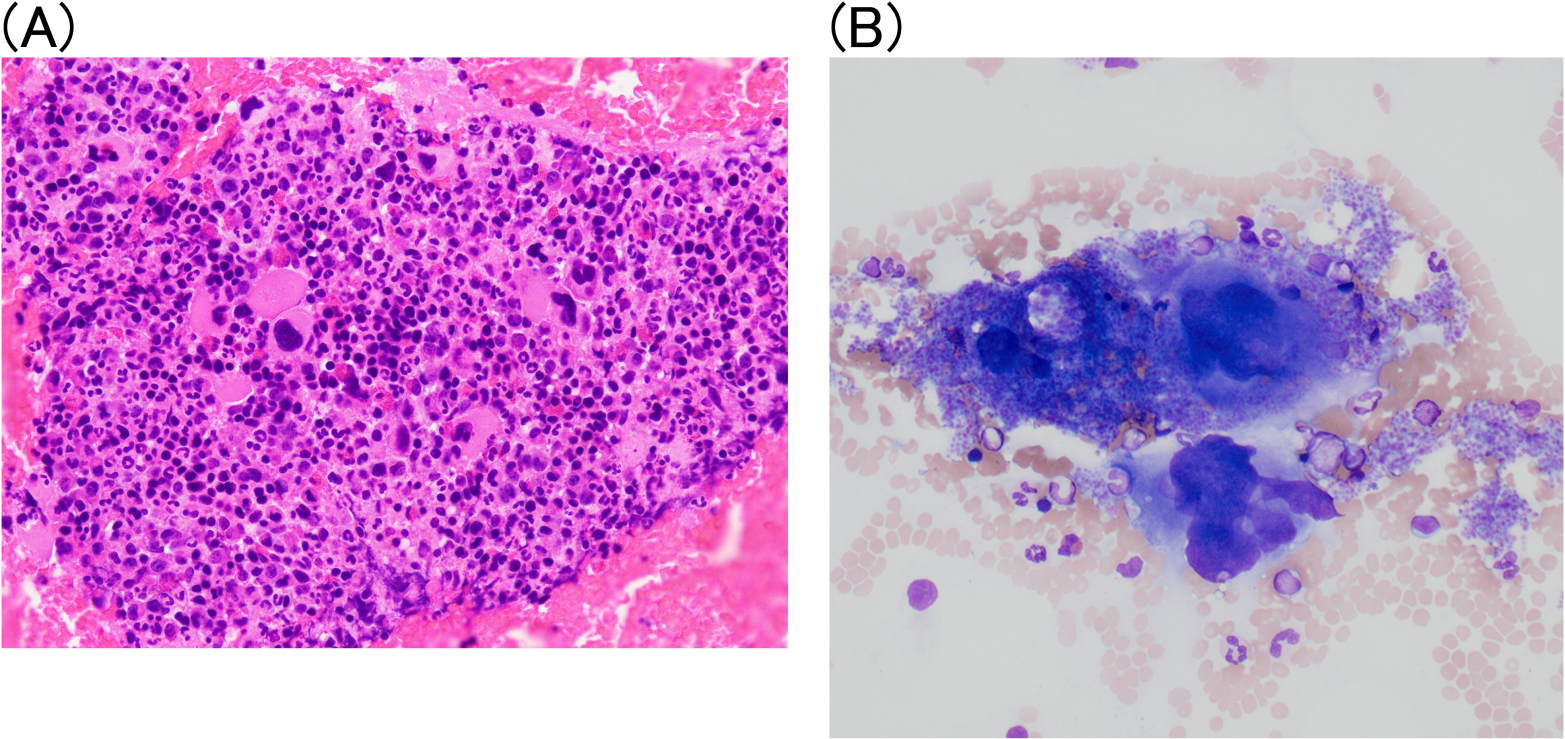
**(A)** Bone marrow findings of *JAK2* V617F–positive essential thrombocythemia. Bone marrow aspiration revealed hypercellularity with megakaryocytic hyperplasia and clustering, consistent with essential thrombocythemia. Original magnification: ×40. **(B)** May–Grünwald–Giemsa stain smear image showed large size and hyperlobulated nuclei. Original magnification: ×200.

**Figure 3:**
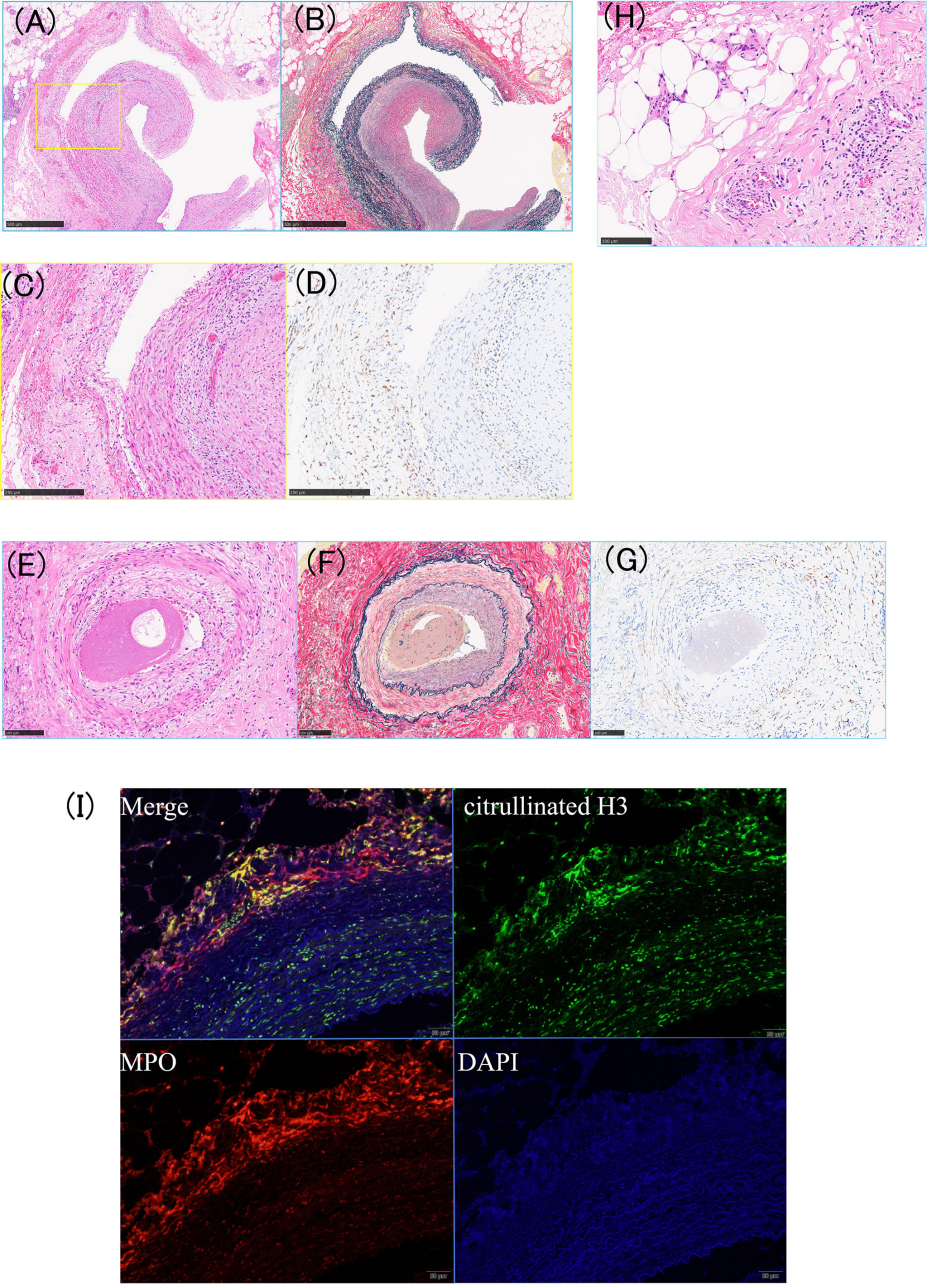
Histopathology of vasculitis and thrombosis in the coronary and internal mammary arteries. **(A–D)** Coronary artery: **(A)** hematoxylin and eosin (HE) staining showing luminal thrombosis and marked intimal thickening, **(B)** Elastica van Gieson (EVG) staining showing disruption or fragmentation of the internal elastic lamina. **(C)** and **(D)** magnify the area outlined by the yellow line in **(A)**. **(C)** moderate inflammatory cell infiltration between the intima and media layers, and **(D)** numerous CD163-positive macrophages within the intima–media layer. **(E–G)** Right internal mammary artery: **(E)** HE staining, **(F)** EVG staining, and **(G)** CD163 immunostaining, each revealing inflammatory infiltration of the vessel wall. **(H)** Capillaries showing neutrophilic infiltration and leukocytoclastic vasculitis. **(I)** Immunohistochemical staining showing neutrophil extracellular trap (NET) formation in the adventitia of the right internal mammary artery, supporting NETosis.

Bone marrow aspiration revealed hyperplastic changes and an increase in the number of CD42b-positive megakaryocytes, characterized by large size and hyperlobulated nuclei (**Figures 2A, B**). Bone marrow biopsy was not performed due to high bleeding risk from triple anticoagulant therapy. Histopathological analysis of the coronary artery and right internal mammary artery revealed thrombosis and intimal thickening, along with CD163-positive cell infiltration into the intima-media and dissection, as well as dissection between elastic fibers in the media (**Figures 3A–G**). Leukocytoclastic vasculitis was observed within the adipose tissue of the right internal thoracic artery specimen (**Figure 3H**). Hypocomplementemic urticarial vasculitis was considered unlikely because C1q immunostaining was negative. IgG4-related disease was also unlikely, as only a few IgG4-positive plasma cells were observed. Infectious vasculitis was excluded by negative cytomegalovirus immunostaining, EBER *in situ* hybridization, and Warthin–Starry staining. Neutrophil infiltration and neutrophil extracellular trap (NET) formation were observed in the vasa vasorum surrounding the right internal thoracic artery (**Figure 3I**).

Based on histopathological infiltration of neutrophils and macrophages into both large and small vessels, the patient was diagnosed with *JAK2* V617F mutation–positive ET complicated by vasculitis.

**Figure 4:**
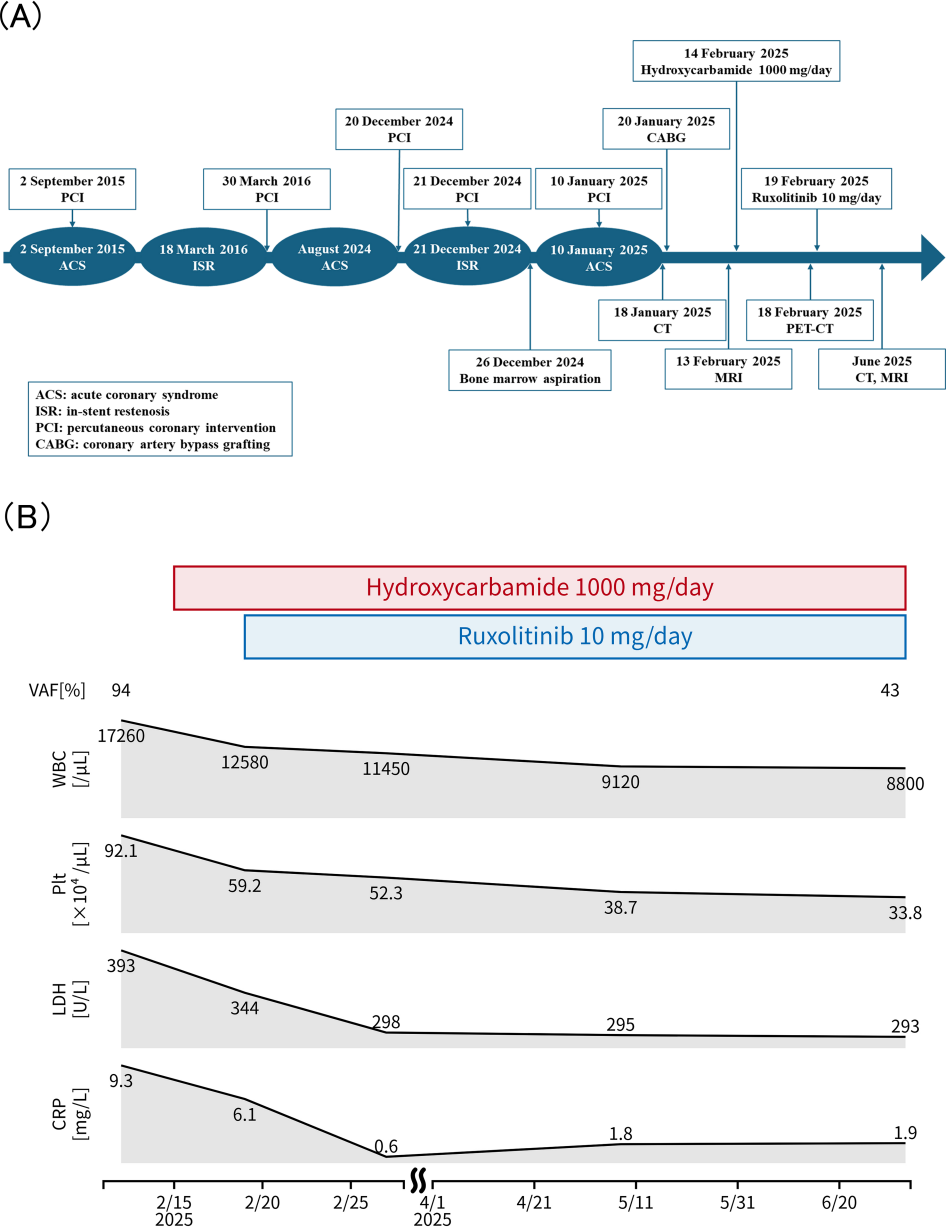
Clinical course and laboratory changes following treatment. **(A)** Timeline of clinical events, including acute coronary syndrome, percutaneous coronary intervention, coronary artery bypass grafting, and diagnostic evaluations. **(B)** Changes in white blood cell count (WBC), platelet count (Plt), lactate dehydrogenase (LDH), C-reactive protein (CRP), and *JAK2* V617F variant allele frequency (VAF) from baseline to 6 months after initiation of hydroxycarbamide and ruxolitinib therapy. *JAK2* VAF was measured from peripheral blood samples.

Considering the recurrence of cardiovascular events, potentially fatal, and the risks of thrombosis and worsening diabetes associated with glucocorticoid use, treatment with ruxolitinib was initiated without glucocorticoids. After treatment with hydroxycarbamide (1, 000 mg/day) and ruxolitinib (10 mg/day), the white blood cell and platelet counts decreased. Computed tomography and magnetic resonance imaging scans performed 6 months after treatment initiation showed an improvement in vascular wall thickening and stenosis (**Figures 1F–H**). The *JAK2* V617F variant allele frequency decreased to 43% (**Figure 4B**). No cardiovascular events occurred.

**Figure 4** illustrates the timeline of events and changes in laboratory data.

## 3 Discussion

This case highlights a rare and intriguing presentation of vasculitis complicating *JAK2* V617F–positive ET, manifesting as recurrent myocardial infarction and coronary stent thrombosis, together with stenotic lesions across arteries of various calibers. Extensive vascular involvement, spanning both large and small vessels, underscores the systemic inflammatory potential of clonal myeloproliferation. Remarkably, treatment with ruxolitinib led to significant clinical improvement, radiologic regression of vascular inflammation, and normalization of platelet counts, suggesting a pivotal role of JAK–STAT pathway inhibition in controlling both vasculitis and myeloproliferation in this case. In this case, recurrent cardiovascular events and stent occlusion suggested a pathophysiology of thrombotic inflammation or vasculitis associated with MPN. An abnormal intraoperative finding of easily dissecting vessel walls was observed, and histological examination confirmed dissection of the vessel wall due to severe inflammatory cell infiltration in the vascular media. These findings support vasculitis as the predominant pathophysiologic mechanism.

Vasculitis associated with hematologic malignancies occurs in 10–25% of cases of conditions such as myelodysplastic syndromes and chronic myelomonocytic leukemia, with approximately one-third presenting with vasculitis-like lesions (5). Large-vessel vasculitis is the most frequent subtype, and *JAK2* V617F–positive MPNs often present with atypical GCA-like findings (5). In *JAK2* V617F–positive MPN with giant cell arteritis, cranial symptoms are infrequent, and steroid dependence, treatment resistance, and reduced overall survival have been reported (5).

*JAK2* gain-of-function mutations activate STAT3/5, promoting immune responses, inflammation, angiogenesis, cell proliferation, and apoptosis suppression (6). Furthermore, as the variant allele frequency increases, it correlates with platelet activation and anti-endothelial cell antibodies, leading to excessive production of inflammatory cytokines and reactive oxygen species from the clonal hematopoiesis (CH) stage (7–9). The presence of CH in peripheral blood cells is associated with atherosclerotic cardiovascular complications, with individuals harboring the *JAK2* V617F mutation exhibiting an increased risk of coronary artery disease and ischemic stroke (10, 11). Multi-omics analyses have revealed that CH alters the immunoreactivity of monocytes and other hematopoietic cells, amplifying proinflammatory signaling (9, 12). MPN patients exhibit elevated levels of various inflammatory cytokines, which correlate with disease activity (13). In this case, the *JAK2* V617F variant allele frequency was markedly elevated, and histopathological examination revealed inflammatory cell infiltration and neutrophil extracellular trap formation in both large and small vessels. This case was ANCA-negative and demonstrated vasculitis involving large vessels, intermuscular arteries, leukocytoclastic vasculitis, and vessels of various sizes. For the differential diagnosis of vasculitis, multiple immunohistochemistry tests were performed, however, no specific results were found. It is considered to be”Vasculitis Associated with Probable Etiology” meeting the Chapel Hill criteria (14), specifically vasculitis triggered by a *JAK2* V617F mutation. Leukocytoclastic vasculitis findings frequently occur in hematologic malignancies (15). Although no skin findings were observed in this case, leukocytoclastic vasculitis was confirmed in the right internal mammary artery biopsy specimen, making this a significant case.

Abnormalities in JAK–STAT signaling have been implicated in GCA and other vasculitis, and histological examination of temporal arteries in GCA revealed IL-6/IL-17A expression and NETosis in the adventitia and vicinity of the vasa vasorum of the temporal artery, with involvement also reported in the development of large vessel vasculitis (16, 17). It has been reported that NETosis is enhanced in *JAK2* V617F mutation-positive MPNs (18–20). NETs are essential for maintaining immune homeostasis, they also activate immune cells such as B cells, antigen-presenting cells, and T cells, contributing to ANCA-associated vasculitis and autoimmune diseases (21). Additionally, the *JAK2* V617F mutation also contributes to increased levels of inflammatory cytokines, such as IL-6, in macrophages (22, 23). These findings suggest that somatic mutations promote a pro-inflammatory environment, accelerating the pathophysiology of vasculitis and thrombosis in MPN patients. Although the gold standard treatment for vasculitis is glucocorticoids, their use increases the risk of recurrent cardiovascular events and can lead to fatal outcomes (24). In a Phase 3 trial of upadacitinib for GCA, improved sustained remission rates and reduced cumulative glucocorticoid use were observed (25). While cardiovascular risk is a concern with JAK inhibitors, no major cardiovascular adverse events occurred in the upadacitinib group for 52 weeks (25). Ruxolitinib, a JAK1/2 inhibitor, suppresses the pathological signaling pathway arising from the *JAK2*-V617F mutation and potentially demonstrates anti-tumor effects through the inhibition of inflammatory cytokine production. Indeed, ruxolitinib is already approved as an immunomodulatory therapy for steroid-refractory graft-versus-host disease (GVHD), exerting anti-inflammatory effects through the suppression of pathogenic cytokine signaling (26). Ruxolitinib reduced plasma levels of numerous inflammatory markers, including IL-6, TNF-α, and C-reactive protein, an acute-phase inflammatory marker (27). Ruxolitinib has also been shown to reduce neutrophil extracellular trap formation in macrophage activation syndrome where the JAK-STAT pathway is activated (28). These data support the efficacy of ruxolitinib in treating vasculitis in this case. JAK inhibitors have been explored as therapeutic options in various forms of vasculitis (29, 30). However, in most reported cases, JAK inhibition was introduced as an anti-inflammatory strategy rather than as targeted suppression of a defined molecular driver. In the present case, vasculitic manifestations improved after initiation of ruxolitinib without the use of glucocorticoids, supporting a mutation-driven inflammatory mechanism. The treatment strategies for MPN-associated vasculitis remain unclear and require further investigation. However, Aldarayseh et al. recently reported the efficacy of ruxolitinib in *JAK2* V617F mutation–positive ET complicated by hydroxycarbamide- and anagrelide-resistant leukocytoclastic vasculitis (31). Hydroxycarbamide reduces thrombotic events; however, it cannot adequately control the underlying pathologies of clonal expansion and chronic inflammation (32). Ruxolitinib may be a treatment that targets the pathophysiology underlying the disease condition, as seen in this case, without the need for concomitant glucocorticoid therapy. Hydroxycarbamide was initiated as the first-line cytoreductive therapy for high-risk essential thrombocythemia. Although hydroxycarbamide resistance was not formally confirmed, persistent vasculitis symptoms and recurrent thrombosis suggested JAK2-driven inflammation, leading to the addition of ruxolitinib.

In this case, improvement in vasculitis lesions was confirmed 6 months after initiating ruxolitinib therapy, and the platelet count normalized. This temporal relationship supports a biologically meaningful association between JAK inhibition and improvement in both vascular inflammation and clonal hematopoiesis. Importantly, ruxolitinib was introduced not solely as a cytoreductive strategy but as targeted modulation of JAK2-driven inflammatory signaling underlying the vasculitis phenotype. Although causality cannot be proven in a single case, the parallel improvement of inflammatory and hematological findings is consistent with a potential role of JAK2-driven signaling in disease activity. However, a key limitation should be acknowledged. Because hydroxycarbamide and ruxolitinib were initiated within a short timeframe, it is not possible to definitively disentangle their respective therapeutic contributions. Although the clinical course suggests a role for JAK inhibition in controlling vasculitis, a direct causal attribution to ruxolitinib alone remains inferential.

## Data Availability

The raw data supporting the conclusions of this article will be made available by the authors, without undue reservation.
